# Ethanol Exposure Alters Protein Expression in a Mouse Model of Fetal Alcohol Spectrum Disorders

**DOI:** 10.1155/2012/867141

**Published:** 2012-06-14

**Authors:** Stephen Mason, Bruce Anthony, Xianyin Lai, Heather N. Ringham, Mu Wang, Frank A. Witzmann, Jin-Sam You, Feng C. Zhou

**Affiliations:** ^1^Department of Anatomy and Cell Biology, Indiana University School of Medicine, Indianapolis, IN 46202, USA; ^2^Department of Cellular and Integrative Physiology, Indiana University School of Medicine, Indianapolis, IN 46202, USA; ^3^Department of Biochemistry and Molecular Biology, Indiana University School of Medicine, Indianapolis, IN 46202, USA; ^4^Monarch LifeSciences, LLC., Indianapolis, IN 46202, USA; ^5^Stark Neuroscience Research Institute, Indiana University School of Medicine, Indianapolis, IN 46202, USA

## Abstract

Alcohol exposure during development can result in variable growth retardation and facial dysmorphology known as fetal alcohol spectrum disorders. Although the mechanisms underlying the disorder are not fully understood, recent progress has been made that alcohol induces aberrant changes in gene expression and in the epigenome of embryos. To inform the gene and epigenetic changes in alcohol-induced teratology, we used whole-embryo culture to identify the alcohol-signature protein profile of neurulating C6 mice. Alcohol-treated and control cultures were homogenized, isoelectrically focused, and loaded for 2D gel electrophoresis. Stained gels were cross matched with analytical software. We identified 40 differentially expressed protein spots (*P* < 0.01), and 9 spots were selected for LC/MS-MS identification. Misregulated proteins include serotransferrin, triosephosphate isomerase and ubiquitin-conjugating enzyme E2 N. Misregulation of serotransferrin and triosephosphate isomerase was confirmed with immunologic analysis. Alteration of proteins with roles in cellular function, cell cycle, and the ubiquitin-proteasome pathway was induced by alcohol. Several misregulated proteins interact with effectors of the NF-**κ**B and Myc transcription factor cascades. Using a whole-embryo culture, we have identified misregulated proteins known to be involved in nervous system development and function.

## 1. Introduction

Fetal alcohol spectrum disorders (FASD) is characterized by a multiorgan phenotype with various degrees of neural developmental deficit, growth retardation, microcephaly, joint deformity, cardiac malformation, and facial dysmorphology due to alcohol exposure during pregnancy [1, 2]. A continuum of deficits affecting multiple aspects of cognition and behavior is seen in FASD-affected children [3]. While the underlying mechanism driving FASD remains unclear [4–6], altered cellular pathways include cellular metabolism [7–9], suppression of protein and DNA synthesis [10], and oxidative stress [11]. Recent evidence suggests alcohol induces aberrant epigenetic changes during neurogenesis, when proper gene and protein expression plays a critical role in neurodevelopment [12]. For example, we have found aberrant DNA methylation results in the misexpression of neurospecification genes *Ngn *and* Sox5 *[12, 13]. Over 2,100 differentially methylated genes have been identified in alcohol-treated embryos, suggesting that multiple cellular pathways are likely to be altered at the protein level [13].

Ethanol has multiple effects on methyl donors and inhibits folate-mediated methionine synthesis [14], thereby interrupting the substrate for biologic methylation. A correlation between altered DNA methylation and the severity of neural tube defects is seen in alcohol-treated embryos [13]. Folate supplementation of FASD models can ameliorate alcohol-induced neural tube defects [15] and protein misregulation in the central nervous system (CNS) [16]. These results suggest methionine synthesis, subsequent DNA methylation, and the resulting epigenome is critical for normal embryonic development [14]. Interestingly, in gravid mice the methionine synthesis pathway is altered with iron deficiency as well as alcohol exposure [17]. Iron deficiency produces CNS defects, such as microencephaly and psychomotor dysfunction, that parallel the defects seen in FASD [18]. Ethanol also affects the intracellular proteolytic pathway, which is essential for cellular regulation and viability [19]. The ubiquitin-proteasome system is the primary cellular pathway for degrading damaged proteins and controlling the level of gene regulatory factors and has been implicated in the etiology of alcoholic liver disease and neurodegenerative disease, including Alzheimer's and Parkinson's disease [20]. Alcohol-fed rodents accumulate protein in the liver, reflecting a decrease in hepatocyte catabolism [19], ostensibly from downregulation of proteasome-interacting proteins and regulatory complexes [21]. More significantly, microarray and proteomic analyses demonstrate reduced expression of proteasome components in alcohol-exposed embryonic mice [16, 22].

Previous gene expression and proteomic analyses of FASD models have been completed, and the results generally overlap. Microarray analysis of alcohol-exposed fetal brain has demonstrated downregulation of genes involved in cell proliferation, differentiation, apoptosis, and the ubiquitin-proteasome pathway [23]. The identified genes have been shown to contribute to tissue growth and remodeling, as well as neuronal growth and survival. Gutala et al. [24] demonstrated that alcohol treatment of mouse cortical cells suppresses the expression of ubiquitin-proteasome and ribosomal pathway genes, leading to disruption of the protein degradation and protein synthesis machinery. Green and colleagues [22] have shown that proteasome and ribosome pathways are downregulated after 3 hours of alcohol exposure at E8; conversely, cellular pathways involved in tight junctions, focal adhesion, and regulation of the actin cytoskeleton are upregulated at 3 hrs. Similarly, proteomic analyses have demonstrated alteration of proteins involved in energy production, cellular signaling, protein translation, and the ubiquitin-proteasome system [16]. Specific proteins known to be alcohol-sensitive include brain-derived neurotrophic factor (BDNF) [25], cellular retinoic acid binding protein I (CRABP-I) [26], and proteasome subunit *α* type-6 [16]. Using embryonic cultures, we have previously found upregulation of cyclin D1 and proapoptotic p53 [27].

In the current study, we have used an established whole-embryo culture model with strictly controlled staging (timed by somite number) and alcohol dose to obtain an alcohol-signature protein profile. This profile may inform the gene and epigenetic changes in alcohol-induced teratology and/or lead to the development of biomarkers of prenatal alcohol exposure. Previous research with this culture model has demonstrated alcohol-induced abnormalities of the neural tube, brain vesicles, optic system, heart, and limb buds [13]. Neural tube abnormalities occur most frequently in the head fold and correlate with more severe genetic and phenotypic abnormalities. To characterize misregulated proteins associated with these developmental abnormalities, we have selected 2D gel electrophoresis to investigate the effects of alcohol exposure on the embryonic proteome. The feasibility of whole-embryo 2D gel electrophoresis has been demonstrated in previous studies [28]. Based on our proteomic and immunologic results, we conclude that the epigenetic and morphologic changes induced by alcohol exposure results in protein misregulation during neurulation.

## 2. Materials and Methods

### 2.1. Embryonic Culture

A whole-embryo culture model was used to study alterations in protein regulation associated with FASD. This FASD model has been described previously by Ogawa et al. [29]. Briefly, C57BL/6 (C6) mice (weighing approximately 20 g) were purchased from Harlan, Inc. (Indianapolis, IN, USA). All animal procedures were approved by the Indiana University Institutional Animal Care and Use Committee. Mouse breeders were individually housed and maintained on a 12 hour light-dark cycle (light on: 19:00–7 : 00) and provided laboratory chow and water ad libitum. When a vaginal plug was detected after the mating period, it was designated as gestational day 0 (GD0) or embryonic day 0 (E0). On E8.25, dams were sacrificed by overdose using CO_2_ gas. The technique for whole-embryo culture was based on the method described by New [30]. Briefly, the gravid uterus was removed and placed in a sterile phosphate buffer containing saline (PBS, 0.1 M) at 37°C. Decidual tissues and the Reichert membrane were removed carefully and immediately immersed in the PBS containing 4% fetal bovine serum (Sigma, St. Louis, MO, USA), leaving the visceral yolk sac and a small piece of the ectoplacental cone intact. Three embryos bearing three to six somites were placed in a culture bottle (20 mL) containing the culture medium, which consisted of 70% immediately centrifuged heat-inactivated rat serum (Harlan Sprague-Dawley, Inc.) and 30% PB1 buffer (137 mM NaCl, 2.7 mM KCl, 0.5 mM MgCl_2_, 8 mM Na_2_HPO_4_, 1.47 mM KH_2_PO_4_, 0.9 mM CaCl_2_, 5.6 mM glucose, and 0.33 mM sodium pyruvate; pH 7.4). The embryos were then supplemented with penicillin and streptomycin (20 units/mL and 20 *μ*g/mL, resp., Sigma). Bottles were gassed at 0 to 22 hr with 5% O_2_, 5% CO_2_, and 90% N_2_ and at 22 to 44 hr with 20% O_2_, 5% CO_2_, and 75% N_2_ in a rotating culture system (B.T.C. Precision Incubator Unit; B.T.C. Engineering, Cambridge, England; 36 rpm) at 37°C. After the preculture period (2–4 hr; maximum, 4 hr), alcohol exposure was started by transferring the embryos into the medium containing 6 *μ*L/mL of 95% ethanol (approximately 400 mg/dL). Before the experiment, the medium alcohol levels in the setting were tested using an Analox alcohol analyzer (Analox Instruments USA, Lunenburg, MA, USA). The control group was cultured in the medium with no ethanol. On day 2, the culture medium was changed 22 hr after the start of the exposure with the same treatments described above. All cultures were terminated 44 hr from the beginning of treatment. A total of 73 embryos from 25 separate cultures were used in this study. In each culture batch, control embryos were included to avoid misinterpretations produced by any differences in the cultural conditions among these experiments.

The concentration of ethanol in the medium across 24 hr was tested at 0, 12, and 22 hr (the timing of medium change), previously, in a separate group not used for vulnerability study [29]. The medium alcohol level used in current study is comparable to the *in vivo* dosage for the lowest teratogenic dose used by Webster et al. [31]. This level is considered within the range attained by human alcoholics [32, 33]. At the end of culture, viability was confirmed by blood circulation of the yolk sac and cardiac activity. Embryos were snap frozen in liquid N_2_.

### 2.2. 2D Gel Analysis

Frozen (−70°C) embryos were pooled (*n* = 7) in a 25 mL beaker, along with 8 volumes of a solution containing 9 M urea, 4% Igepal CA-630 ([octylphenoxy] polyethoxyethanol), 1% dithiothreitol (DTT), and 2% carrier ampholytes (pH 8–10.5), and thoroughly sonicated. After solubilization at room temperature, samples were centrifuged at 100,000 × g for 30 min using a Beckman TL-100 ultracentrifuge (Fullerton, CA, USA) to remove nucleic acid and insoluble materials; the supernatants were stored at −45°C until 2DE separation. Using overnight, passive rehydration at room temperature, homogenized sample was loaded onto immobilized pH-gradient (IPG) strips (24 cm, nonlinear pH 3–10: BioRad: Hercules, CA, USA). Isoelectric focusing (IEF) was performed with a Protean IEF Cells (BioRad) (10 strips/instrument), by a program of progressively increasing voltage for a total of 100,000 Vhr. A computer-controlled gradient casting system was used to prepare second-dimension (2D) sodium dodecyl sulfate (SDS) gradient slab gels (20 × 25 × 0.15 cm) in which the acrylamide concentration varied linearly from 11% to 17% T. First-dimension IPG strips were loaded directly onto the slab gels following equilibration for 10 min in buffer I. All nine 2D slab gels were run in parallel at 8°C for 18 h at 160 V and subsequently fixed and stained using a sensitive colloidal Coomassie Blue G-250 procedure [34]. After 96 h, gels were washed several times with water and scanned at 95.3 *μ*m/pixel resolution using a GS-800 Calibrated Imaging Densitometer (Bio-Rad). The resulting 12 bit images were analyzed using PDQuest software (Bio-Rad, v.7.1). Background was subtracted and protein spot density peaks detected and counted. Because total spot counts and the total optical density are directly related to the total protein concentration, individual protein quantities were thus expressed as parts-per-million (ppm) of the total integrated optical density, after normalization against total image density. A reference pattern was constructed and each of the gels in the match set was matched to the reference gel. Numerous proteins that were uniformly expressed in all patterns were used as landmarks to facilitate rapid gel matching. Protein spots that were not present in at least 3 of the gels for both groups were removed from the analyses. A statistical mean was determined for each spot in both the alcohol-treated and control groups. A *t*-test was performed between the two groups to determine which spots had a significantly different staining intensity (*P* < 0.01). Protein spots were excised from the gels and destained with acetonitrile (ACN) in 50 mM NH_4_HCO_3_, reduced with 10 mM DTT, alkylated with iodoacetamide, and in-gel digested with modified trypsin at 37°C overnight, followed by serial extraction with ACN. Tryptic peptides were analyzed on a LTQ mass spectrometer (Thermo Electron, San Jose, CA, USA) coupled with an Agilent 1100 HPLC system (Agilent Technologies, Santa Clara, USA) and C18 nanocolumn (75 *μ*m × 5 cm). Peptides were eluted with a linear gradient from 5 to 45% acetonitrile developed over 60 min at a flow rate of 300 nL/min. The acquired MS/MS data were filtered and analyzed by a proprietary algorithm [35]. Database searches against the IPI (International Protein Index) mouse database and the nonredundant mouse database were carried out using both the X!Tandem and SEQUEST algorithms.

### 2.3. Ingenuity Pathways Analysis

Identified proteins were submitted to Ingenuity Pathways Analysis (IPA) (Ingenuity, Inc. http://www.ingenuity.com) online software. IPA builds networks based upon information extracted from the scientific literature and deposited in the Ingenuity Knowledge Base. IPA provides an assessment of the signaling and metabolic pathways, molecular networks, and biological processes that are most significantly perturbed in the experimental condition. The Path Designer feature was utilized to abridge the molecular network for publication.

### 2.4. Western Blot Analysis

Western blotting was used to confirm the misregulation of proteins of mechanistic interest in FASD, as previously described [27]. For total protein procurement, 3 embryos were pooled as one sample. Homogenized embryos were loaded on two, 8–12% PAGE gels. Proteins were transferred to a nitrocellulose membrane and blocked overnight with 5% milk and 0.02% BSA, washed 3× in TBST, then incubated for 2–4 h at 30°C using the appropriate 1° antibody. Primary antibodies included: goat poly (rC)-binding protein 1, rabbit serotransferrin and triosephosphate isomerase (Santa Cruz Biotechnology), and mouse Ube2N (Abcam). Monoclonal anti-GAPDH-peroxidase (Sigma) (1 : 40,000 dilution) was incubated as internal control for densitometry. Membranes are washed and incubated with the appropriate HRP conjugated 2° antibody and detected with a Pierce ECL detection kit (Thermo Fisher Scientific, Rockford, IL, USA). Blots are detected with a FOTO/Analyst Luminary/FX system (FOTODYNE Inc., Hartland, WI, USA), and densitometric comparisons were made with TotalLab Quant software (v.11.5). GAPDH density measurements were used for load controls, and variations in measurements were adjusted by percentage to the highest values for that membrane. Measurements for each target protein were then adjusted in accordance with the GAPDH load controls. All changes in protein expression from alcohol treatment are reported as a percentage change compared to controls, with a minimum of 3 samples/treatment group. Statistical analysis used Student's *t*-test on Microsoft Excel (v. 14).

### 2.5. Immunohistochemistry Analysis

The embryos of the two treatment groups (alcohol = 4, control = 4 embryos) were paired and embedded in gelatin together at E10.25, with careful alignment of their level and orientation. They were sectioned in 40 micron and processed for immunohistochemistry in parallel; this practice avoids any bias through the staining procedure and is convenient for comparing levels of embryo sections side by side. All sections were then washed 3× in PBS. They were then incubated in (1) 3% H_2_O_2_ (v/v) in PBS for 30 minutes and (2) PBS containing 4% normal control serum, and 0.3% Triton X-100 (TX) for 30 minutes at room temperature to block nonspecific binding. Sections were next incubated with antibodies against serotransferrin (1 : 50) and triosephosphate isomerase (1 : 50). All 1° antibodies were incubated overnight at room temperature. The sections were then incubated with a 2° antibody (1 : 250, Jackson ImmunoResearch Laboratory, West Grove, PA) for 1 hr, followed by an avidin-biotin peroxidase complex (Vector Laboratories) for 1 hr at room temperature. All sections were washed 3× with PBS for 5 minutes between each antibody incubation. The color reaction (brown) was developed by adding 0.05% 3′ to 3′-diaminobenzidine tetrahydrochloride and 0.003% H_2_O_2 _ in Tris buffer to reveal the peroxidase activity.

## 3. Results

### 3.1. Embryonic Growth Retardation/Abnormalities

**Figure 1:**
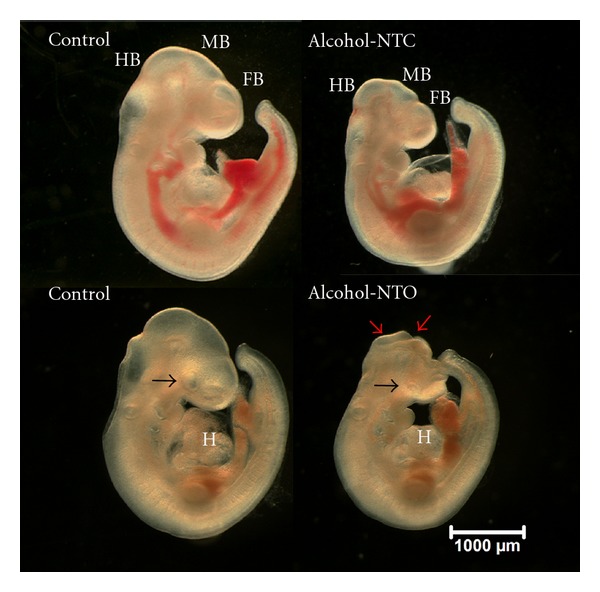
Alcohol-induced dysmorphology of embryonic development at E10.25. The alcohol-treated embryos (right side of figure) showed developmental delay and abnormalities. Examples are demonstrated in small forebrain (FB), midbrain (MB), or hindbrain (HB); abnormality of the heart (H); small eyes (black arrows) as compared with control (left). A reduced blood/vascular system with lower red coloration was apparent in the alcohol-treated embryos. A major dichotomic phenotype is embryos with neural tube closed but with delay and abnormality in development (Alcohol-NTC), and neural tube opened (Alcohol-NTO). An open neural tube is shown (red arrows) in an alcohol-treated embryo (bottom right). Scale bar: 1,000 micron.

Among all cultured embryos, more than 95% maintained active heartbeats and blood circulation during the incubation period, and only those were used for analysis. Control and alcohol-treated embryos are shown in Figure 1. Embryonic development was significantly compromised by alcohol-exposure, consistent with previous studies [12, 13, 36, 37]. Growth retardation and abnormalities of the brain, heart, and limbs were seen in alcohol-treated embryos. Specific abnormalities include neural tube defects, heart malformations, small/slanted eyes, abnormal tail morphology, and unfinished turning of the neural axis. A reduced blood/vascular system with less yolk sac vascularity and a lower red coloration was apparent in the alcohol-treated versus control embryos.

### 3.2. 2D Gel Analysis

**Figure 2:**
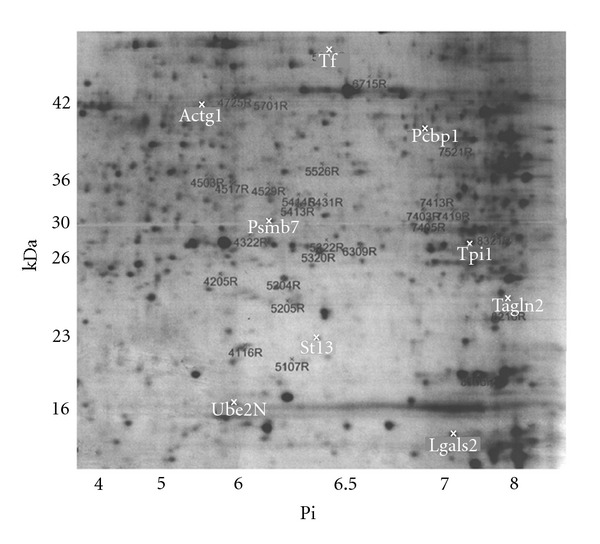
Sample 2D gel separation of whole-embryo proteins. Whole-embryos were solubilized and the proteins separated using IPG strips in the 1st dimension and SDS gradient slab gels in the 2nd dimension. The protein profiles were visualized by Coomassie blue staining. Nine MS/MS identified protein spots with differential expression due to alcohol treatment are highlighted with a white “x” and labeled with the gene name. The remaining spots with altered expression are labeled with the spot number in black font followed by “R,” signifying this gel as the reference (R) control.

The total number of gel spots detected by PDQuest was 2,050. Forty gel spots with significant (*P* < 0.01) differential expression (>1.5-fold change) between alcohol-treated and control embryos were identified. Integrated densities of 8 gel spots fell below 200 ppm, a protein density that is too low for reliable MS/MS identification. Of the remaining 32 spots, 24 were downregulated and 8 were upregulated in alcohol-treated embryos. To prioritize the study, only those spots with a highly significant (*P* < 0.007) difference in expression levels were selected for analysis. Five downregulated and 4 upregulated spots were selected for LC-MS/MS identification (Figure 2).  Table 1 lists the numerical assignment, number of peptides used for identification, UniProtKB accession number, gene name, and the protein identification for each spot with a significant (*P* < 0.007) difference in expression levels between the two groups.

### 3.3. Western Blot Analysis

**Figure 3:**
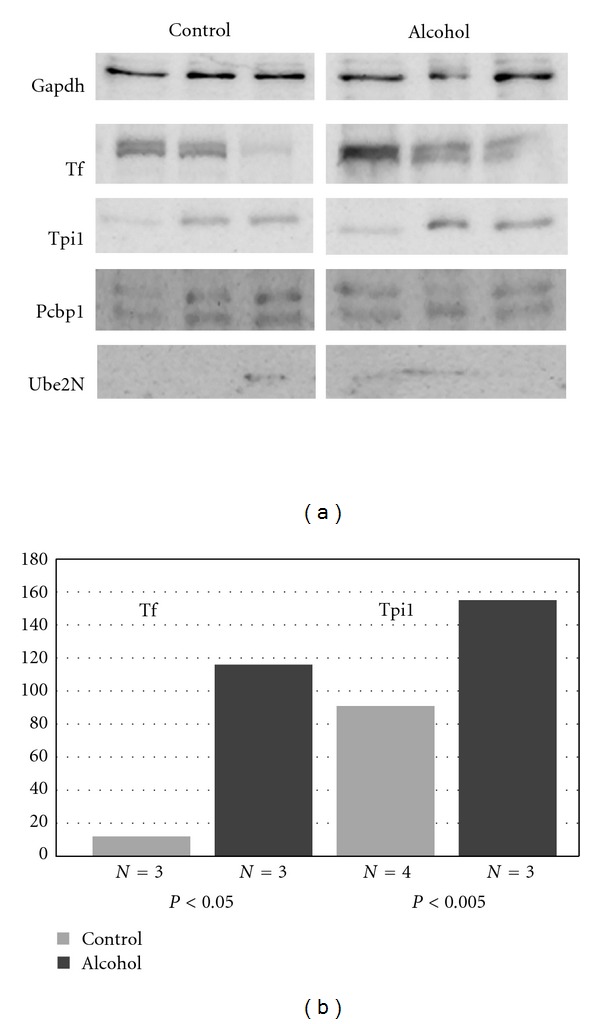
Misregulated proteins of mechanistic interest in FASD were confirmed with Western blotting. Both alcohol and control protein (20 *μ*g) samples were run on SDS-PAGE gels and detected by chemical luminescence. GAPDH was used as an internal control. Statistical analysis of band density by Student's *t*-test showed that alcohol upregulates serotransferrin (Tf) and triosephosphate isomerase (Tpi1) (*P* ≤ 0.05).

Western blot image density analysis confirmed upregulation of serotransferrin (Tf) and triosephosphate isomerase (Tpi1) (*P* < 0.05) (Figure 3). Poly (rC)-binding protein 1 (Pcbp1) and ubiquitin-conjugating enzyme E2 N (Ube2N) are downregulated on 2D gel analysis, but this was not statistically significant by Western blot densitometry. The Ube2n band is faint.

### 3.4. Immunohistochemistry

**Figure 4:**
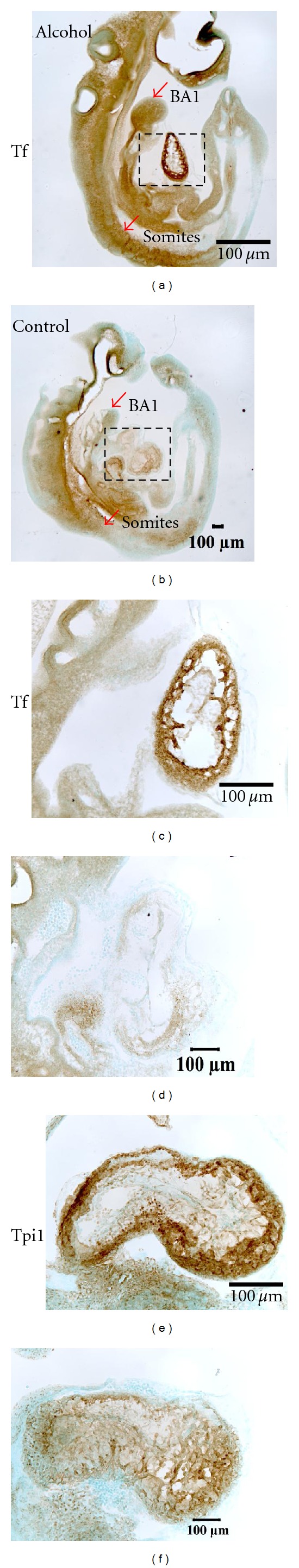
Immunolocalization of serotransferrin to the heart, branchial arches and somites with alcohol treatment. Serotransferrin immunostaining (Tf-im) (a,b,c,d) and triosephosphate isomerase immunostaining (Tpi1-im) (e,f) are increased in alcohol-treated embryos at E10.25. Tf-im consistently localized to the heart (dotted region), branchial arch 1 (BA1) and somites (somites) of alcohol-treated embryos (a) relative to control (b). High magnification of the dotted region is shown in (c) (Alcohol) and (d) (Control). Tpi1-im was diffusely increased in alcohol-treated embryos (e) relative to control (f), but the staining pattern was less consistent.

Immunostaining (im) of E10.25 embryos demonstrated upregulation of Tf and Tpi1 in alcohol-treated embryos. Tf-im consistently localized to the heart, branchial arches and somites of treated embryos relative to control (Figure 4). Tpi1-im was diffusely increased; however, this staining pattern was less consistent among treated embryos.

### 3.5. Ingenuity Pathways Analysis

**Figure 5:**
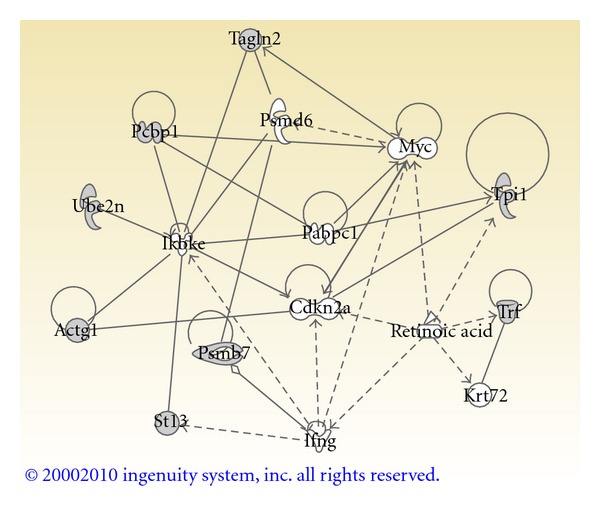
Ingenuity Pathways Analysis of misregulated proteins from alcohol-treated whole-embryo culture. Proteins with altered regulation were analyzed with Ingenuity Pathways Analysis online software. This Core Analysis highlights the signaling and metabolic pathways, molecular networks, and biological processes that are most significantly perturbed in the alcohol-treated cultures. The Ingenuity Path Designer feature was utilized to abridge the molecular network. The proteins are represented by shapes that reflect protein function (https://analysis.ingenuity.com/pa/info/help/help.htm#legend.htm) and labeled with their gene name. Proteins identified in alcohol-treated embryos have shaded shapes; molecules not identified in this study have clear shapes. These molecules were included in the abridged network if they were linked to multiple identified proteins or formed integral links between identified proteins. Solid lines indicate direct interactions and dashed lines are indirect interactions. Abbreviations: Actg1—Gamma actin; Cdkn2a—Cyclin-dependent kinase inhibitor 2A; Ifng—Interferon gamma; Ikbke—Inhibitor of kappa b kinase epsilon; Krt72—Keratin 72; Myc —v-myc myelocytomatosis viral oncogene homolog (avian); Pabpc1—poly(A) binding protein, cytoplasmic 1; Pcbp1—poly(rC) binding protein 1; Psmb7—Proteasome subunit *β* type 7; Psmd6—Proteasome 26S subunit, non-ATPase, 6; St13— Hsp70 interacting protein; Tagln2—Transgelin 2; Trf (Tf)—Serotransferrin; Tpi1—Triosephosphate isomerase 1; Ube2n—Ubiquitin-conjugating enzyme E2N.

Nine proteins identified by LC-MS/MS were submitted to IPA to assess the biological networks perturbed by alcohol treatment (Figure 5). The top reported networks were cell cycle, cell death, gene expression and reproductive system development and function. The top reported biological functions were antigen presentation, cell cycle, cellular signaling, cellular function and maintenance, hematopoiesis, and immune cell trafficking. The top canonical pathways affected by alcohol treatment are clathrin-mediated endocytosis signaling and the ubiquitin-proteasome pathway.

## 4. Discussion

This study was designed to characterize the alcohol-signature protein profile of a whole-embryo culture model of FASD. The whole-embryo was studied at an early, neurulating stage, and the resulting protein profile represents an average across all cell types and tissues. While the embryo system is less sensitive for identifying misregulated proteins in individual organs, our results clearly demonstrate that this model is sensitive for detecting systemic protein misregulation. The analysis detected 40 differentially expressed protein spots, and 9 spots were identified by MS/MS. The possibility exists that some of these protein expression changes may be posttranslational modifications (PTM); nevertheless, these PTM might be important to the pathology of FASD and could be characterized in subsequent studies.

The abnormal embryonic development resulting from the alcohol treatment is consistent with our previous report [29]. The affected structures were derived from each of the three germ layers and involve a wide range of tissues and organs. These effects on protein regulation may parallel the growth delay and developmental abnormalities including brain, neural tube, eye, heart, blood cells, and embryonic vascularization, which are major targets in FASD [13].

As explained in the methods section, 9 differentially expressed proteins were submitted for Ingenuity analysis. Perturbations in cellular function and maintenance, cell cycle, and the ubiquitin-proteasome pathway were demonstrated by Ingenuity. Biological functions altered by alcohol include hematopoiesis and clathrin-mediated endocytosis. We have previously confirmed absent expression of 4 key hematopoietic genes (*Add2*, *B2m*, *Cp*, and *Gpa*), along with an embryonic phenotype consistent with reduced hematopoiesis in alcohol-exposed embryos [12]. Previous studies have demonstrated perturbations of clathrin-mediated endocytosis in adult rat brain extracts [38]; this may result in alterations of postsynaptic plasticity. The Ingenuity molecular network (Figure 5) suggests that the NF-*κ*B cascade and transcription factor Myc may be specifically involved in the pathogenesis of FASD. Five ethanol sensitive proteins interact with Protein IKBKE, a kinase that phosphorylates inhibitors of NF-*κ*B and leads to NF-*κ*B activation [39]. The transcription factor NF-*κ*B protects cells against DNA damage-induced cell death and may play a role in the immune response. Alcohol differentially regulates NF-*κ*B activity in various tissues [40]. Ethanol activates NF-*κ*B in cerebral vascular muscle cells [41], in contrast, ethanol diminishes NF-*κ*B target gene expression in fetal rhombencephalic neurons via a 5-HT(1A) (serotonin) mediated pathway [42]. Ethanol also interferes with inflammatory cytokine production by downregulating NF-*κ*B in monocytes, resulting in impaired immunity [42, 43]. The transcription factor Myc activates growth-related genes [39]. Figure 5 shows that Pcbp1 and Tagln2 directly bind with Myc. Ethanol can induce release of intracellular calcium that stimulates the cell cycle and the expression of Myc proteins associated with cell proliferation. Increased proliferation is advantageous during reimplantation, but ethanol interference with terminal differentiation during organogenesis may underlie alcohol teratogenicity [44].

Serotransferrin (Tf) is upregulated by proteomic and immunologic analysis (Figures 2–4), which may be a compensatory response to the altered iron regulation seen with alcohol exposure. Tf transports iron from tissue sites of absorption to sites of storage or utilization and has a role in stimulating cell proliferation [39]. Because iron is an essential cofactor in neurotransmitter synthesis and myelination, maternal iron status is an important modulator of fetal alcohol-induced neurobehavioral damage [45, 46]. Interestingly, fetal alcohol syndrome produces defects that parallel the abnormalities of early iron deficiency, including microcephaly and motor and behavioral deficits [18]. Our observation that Tf localizes to the heart, branchial arches and somites with alcohol treatment is interesting (Figure 4). These findings warrant further investigation into the association between altered iron regulation, cardiac malformation, and FASD.

Protein triosephosphate isomerase (Tpi1) and transgelin (Tagln2) were upregulated in our proteomic analysis. Tpi1 misregulation was confirmed with Western blot. Diffuse Tpi1-im was seen on whole-embryo immunohistochemistry (Figure 4). Our previous proteomic analysis demonstrated upregulation of Tpi1 in the nucleus accumbens of alcohol-exposed adult rats [47]. Green et al. noted upregulation of pentose phosphate pathway genes in alcohol-exposed C6 mouse embryos [22]. Tpi1 is an enzyme of the pentose phosphate pathway that binds to microtubules and other subcellular components. Protein Tpi1 can reduce the enzymatic activity of other metabolic proteins when bound to them [48]. Tagln2 is one of the earliest markers of differentiated smooth muscle. The function of Tagln2 has not yet been determined, but proteomic analysis has demonstrated altered regulation during neurulation [49, 50]. 

The protein ubiquitination pathway targets molecules for proteasomal degradation through conjugation with Lys48-linked ubiquitin. Alternatively, proteins modified with Lys63-linked ubiquitin chains play roles in signal transduction through the NF-*κ*B cascade, receptor endocytosis, and DNA-repair processes [51]. Degradation of damaged proteins is critical for cellular viability, and evidence suggests ethanol can downregulate components of the ubiquitin-proteasome system [19]. Previous studies have shown that proteasome *β* type-7 is downregulated at the gene and protein level in a murine model of FASD [16, 22]. Our 2D gel analysis also demonstrates downregulation of proteasome *β* type-7. This proteasome subunit forms the catalytic core of the 26S proteasome [19], the main mechanistic component of intracellular proteolysis.

Our 2D gel analysis demonstrated downregulation of ubiquitin-conjugating enzyme E2 N (Ube2N) with alcohol-exposure, congruent with our previous analysis of adult rat brain extracts [47]. A gene expression analysis demonstrated that *Ube2b*, a member of the ubiquitin-conjugating enzyme family, was downregulated in neurulating embryos [12]. Ube2N and Ube2b both catalyze the synthesis of Lys63-linked poly-ubiquitin chains and are involved in the postreplication repair of damaged DNA. Interestingly, a Drosophila homolog of Ube2N was originally isolated as bendless protein, whose mutation affects neuronal synaptic connectivity and visual system development [52–54]. As shown in Figure 5, Ube2N binds with inhibitor of nuclear factor kappa-B kinase subunit *ε* (Protein IKBKE), a kinase that activates the NF-*κ*B pathway. The Western blot analysis demonstrated a faint band at the expected molecular weight of Ube2N (~17 kDa) (Figure 3). *Ube2N* gene expression is temporally regulated during development [55], and our results suggest that Ube2N protein is only marginally expressed at E10.

Hypoxia and ischemia regulate the expression of several important genes at the level of transcription and of mRNA stability. An isoform of poly (rC)-binding protein (Protein Pcbp1), previously identified as an RNA-binding protein, binds to a hypoxia-inducible protein-binding site in the 3′ region of erythropoietin mRNA and regulates stability [56, 57]. Protein Pcbp1 is downregulated in our proteomic analysis; however, Western blot did not demonstrate significant misregulation. The embryonic phenotype is consistent with reduced hematopoiesis in this FASD model [12]. Since Pcbp1 protects erythropoietin mRNA, we hypothesize that downregulation of Pcbp1 may be the mechanism for decreased hematopoiesis.

Galectin-2, which is expressed in differentiated enteric tissue [58], is downregulated in our analysis. Previous experiments in Xenopus embryos have shown that alcohol exposure causes fetal enteric damage and reduced body length [59]. We hypothesize that downregulation of galectin-2 in our FASD model reflects gut underdevelopment.

In summary, alcohol exposure during the period of early neurulation results in an alcohol-signature protein profile. This profile should contribute to (1) generation of testable new hypotheses concerning the mechanistic pathway from protein misregulation to alcohol-induced teratogenesis and (2) development of clinical biomarkers of prenatal alcohol exposure. We identified proteins (serotransferrin, Ube2N) known to be involved in development of the nervous system, and that the NF-*κ*B cascade and the transcription factor Myc may be specifically involved in alcohol-induced teratology. These misregulated proteins may reflect altered cellular pathways secondary to epigenetic, posttranscriptional, or morphologic changes.

## Figures and Tables

**Table 1:** Protein identification of differentially expressed spots from 2D gel analysis of alcohol-treated embryos. The spot assignments, number of peptides used for identification, UniProtKB accession number, gene name, and the protein identification for each spot. Proteins are grouped by their pattern of expression (up- or downregulated) in alcohol-treated embryos relative to control.

Spot ID	No. peptides	Accession	Gene ID	Protein ID
Proteins upregulated in alcohol-treated embryos				
5829	54	Q921I1	*Tf*	Serotransferrin precursor
8217	9	Q9WVA4	*Tagln2*	Transgelin-2
4601	8	Q9QZ83	*Actg1*	Gamma actin-like protein
7324	5	P17751	*Tpi1*	Triosephosphate isomerase

Proteins downregulated in alcohol-treated embryos				
7605	54	P60335	*Pcbp1*	Poly (rC)-binding protein 1
4424	36	P70195	*Psmb7 *	Proteasome subunit *β* type-7
4110	21	P61089	*Ube2n *	Ubiquitin-conjugating enzyme E2 N
7013	16	Q9CQW5	*Lgals2*	Galectin-2
5112	7	Q99L47	*St13*	Hsc70-interacting protein

## Abbreviations

2D: Two-dimensional
ACN: Acetonitrile
BDNF: Brain-derived neurotrophic factor
BSA: Bovine serum albumin
CNS: Central nervous system
CRABP-I: Cellular retinoic acid binding protein I
DTT: Dithiothreitol
FASD: Fetal alcohol spectrum disorder
HRP: Horse radish peroxidase
im: Immunostaining
IPA: Ingenuity Pathways Analysis
IPG: Immobilized pH-gradient
IPI: International Protein Index
IEF: Isoelectric focusing
LC-MS/MS: Liquid chromatography-tandem mass spectrometry
PTM: Post-translational modification
TBST: Tris-buffered saline with Tween.
